# Liquid Chromatography Quadrupole Time-of-Flight Tandem Mass Spectrometry Characterization of Ethyl Acetate Fraction from *Sargassum pallidum* and Its Anti-Melanogenesis Effect in B16F10 Melanoma Cells and Zebrafish Model

**DOI:** 10.3390/ijms26041522

**Published:** 2025-02-11

**Authors:** Wook-Chul Kim, Hyeon Kang, Seung-Hong Lee

**Affiliations:** 1 Department of Medical Science, Soonchunhyang University, Asan 31538, Republic of Korea; wookchul0828@naver.com (W.-C.K.); rkgns21@naver.com (H.K.); 2 Department of Pharmaceutical Engineering, Soonchunhyang University, Asan 31538, Republic of Korea

**Keywords:** melanogenesis, *Sargassum pallidum*, ethyl acetate fraction, B16F10 melanoma cells, zebrafish

## Abstract

Melanin overproduction causes various skin diseases, such as spots, freckles, and wrinkles, resulting in the requirement of melanin synthesis inhibitors like 1-phenyl-2-thiourea (PTU) and kojic acid, which have been commonly used in the pharmaceutical industry. However, these inhibitors can cause side effects such as skin irritation and allergies. Therefore, it is necessary to develop safe and effective melanin inhibitors from natural resources. The purpose of this study was to investigate a whitening agent from natural substances using B16F10 melanoma cells and zebrafish model. We investigated the melanogenesis-inhibiting activities of the fractions from *Sargassum pallidum* extract. The ethyl acetate fraction from *S. pallidum* extract (SPEF) significantly decreased tyrosinase activity. SPEF also significantly reduced α-melanocyte stimulating hormone (MSH)-induced intracellular tyrosinase activity and melanin content in B16F10 cells. Moreover, SPEF inhibited the expression levels of key melanogenic proteins such as tyrosinase, TRP-1, TRP-2, and MITF by downregulating the phosphorylation levels of CREB and PKA in α-MSH-stimulated melanoma cells. Furthermore, SPEF significantly suppressed melanin synthesis in the zebrafish model with no developmental toxicity. LC-Q-TOF-MS/MS analysis identified that SPEF was composed of 12 phytochemical compounds, including diterpenes, which were the dominant metabolites. These results altogether show that SPEF effectively suppresses melanogenesis in B16F10 melanoma cells and in a zebrafish model, with potential for usage in pharmaceuticals and cosmeceuticals.

## 1. Introduction

Melanin, known as an important biomolecule in the defensive mechanism of the human body, protects the skin from ultraviolet (UV) radiation. Melanocytes, specialized cells situated in the stratum basale, which is the deepest layer of the epidermis, are responsible for melanin production via a complex physiological process [1,2]. The melanin synthesis process is promoted by several key melanogenesis-related proteins, including tyrosinase, microphthalmia-associated transcription factor (MITF), tyrosinase-related protein-1 (TRP-1), and tyrosinase-related protein-2 (TRP-2) [3]. Furthermore, cAMP response element-binding protein (CREB), which is phosphorylated and activated by protein kinase A (PKA) in response to elevated intracellular cAMP levels, mediates downstream gene expression. PKA-induced CREB activation facilitates microphthalmia-associated transcription factor (MITF) expression, a dominant regulator of melanin synthesis process [4,5,6].

However, melanin overproduction can trigger a wide range of skin diseases, including hyperpigmentation, freckles, and age-related dark spots. Skin-specific melanin accumulation can contribute to an uneven skin tone and an aged skin phenotype. These pigmentation issues not only affect the aesthetic appearance of the skin but can also have psychological impacts, leading to a higher demand for effective skin-whitening and depigmentation treatments [7]. Propylthiouracil (PTU) and arbutin are classic skin- whitening agents used in the pharmaceutical and cosmetics industries. However, many side effects, including allergic skin responses and irritation, have been reported. In particular, PTU has been associated with side effects such as hair loss, sialadenitis, myopathy, and abnormalities [8,9]. Therefore, there has been a growing need to develop safe and effective anti-melanogenic materials [10].

Living organisms produce natural compounds or substances which encompass a wide variety of secondary metabolites. These metabolites are classified according to their chemical structure and composition, including phenolic, terpenoid, and alkaloid compounds. These compounds have revealed many pharmacological effects, such as antioxidant, anti-inflammatory, anticancer, and antibacterial properties [11]. In particular, numerous studies have reported that brown algae are rich sources of various active compounds. Many previous studies have shown multiple beneficial effects of active components derived from brown algae on human health [12]. Additionally, brown algae and their active compounds have been shown to improve skin health via the promotion of moisturizing, inhibition of wrinkle generation, and whitening. Therefore, brown algae and components derived from them can be utilized as potent pharmacological and cosmeceutical candidates [10].

*Sargassum pallidum*, a type of marine brown algae, is found in the coastal regions of Korea (particularly the southern coast and Jeju Island), China, Japan, and Indonesia. *S. pallidum* is known to contain significant amounts of bioactive compounds, including phenolics, fatty acids, and polysaccharides, which contribute to its diverse pharmacological properties. Many studies have reported that these compounds exhibit strong antioxidant, anti-inflammatory, anticancer, and hypoglycemic activities [13,14]. Interestingly, our previous study has demonstrated strong antioxidant and anti-inflammatory effects of the ethyl acetate fraction derived from *S. pallidum* extract (SPEF) on particulate matter (PM)-induced skin cells [13,15]. These properties sufficiently elucidate that SPEF can be a safe and effective natural skin-brightening agent, which contributes to the alleviation of skin irritation and the reduction in inflammatory hyperpigmentation as an alternative to classic melanin inhibitors, i.e., PTU and arbutin [16].

Hence, our present study investigated the anti-melanogenesis effects of SPEF in α-melanocyte stimulating hormone (MSH)-induced B16F10 melanoma cells and zebrafish model. We also profiled the components of SPEF using the LC-Q-TOF MS/MS method to portray its skin-brightening activities. SPEF shows potential as a safe and effective depigmenting agent in both pharmaceutical and cosmeceutical applications.

## 2. Results

### 2.1. Anti-Tyrosinase Activities of the Fractions of S. pallidum Extract

The 70% ethanol extract of *S. pallidum* (SPE) was dissolved in distilled water and fractionated to the hexane fraction (SPHF), chloroform fraction (SPCF), ethyl acetate fraction (SPEF), butanol fraction (SPBF), and water fraction (SPWF) using solvent–solvent partitioning. The tyrosinase inhibition activities of SPE and its fractions were determined by calculating the concentration that inhibits the reaction by 50% (IC_50_ in µg/mL) (Table 1). These results indicate that SPEF shows the highest tyrosinase-inhibitory activity between the extract and fractions, and subsequent experiments in B16F10 melanoma cells and zebrafish model were conducted using SPEF.

### 2.2. Effects of SPEF on Cytotoxicity in B16F10 Cells

**Figure 1:**
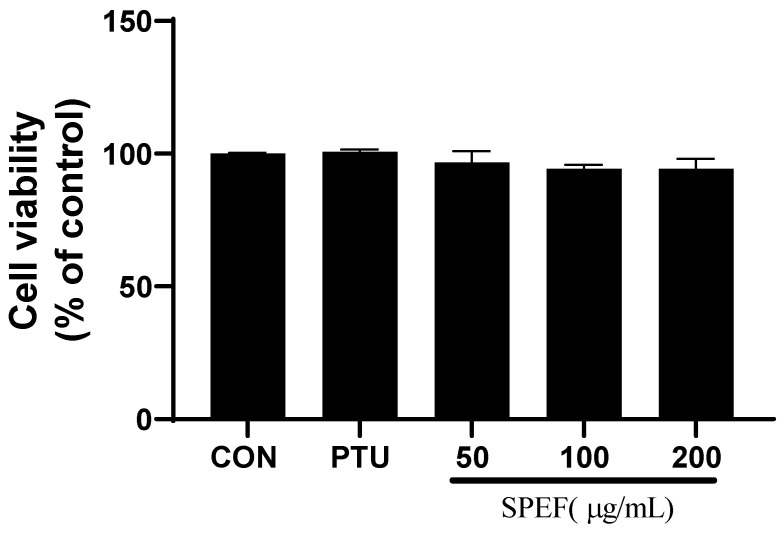
Effects of SPEF and 1-phenyl-2-thiourea (PTU) on cell viability in B16F10 cells.

We first examined the effects of SPEF on the cell viability of B16F10 cells using MTT assay to determine the concentration of SPEF in cell treatment. As shown in Figure 1, SPEF (50–200 μg/mL) and PTU (0.2 mM) treatment for 72 h had no significant cytotoxicity in B16F10 melanoma cells.

### 2.3. Effects of SPEF on Melanin Synthesis and Intracellular Tyrosinase Activity in B16F10 Cells

**Figure 2:**
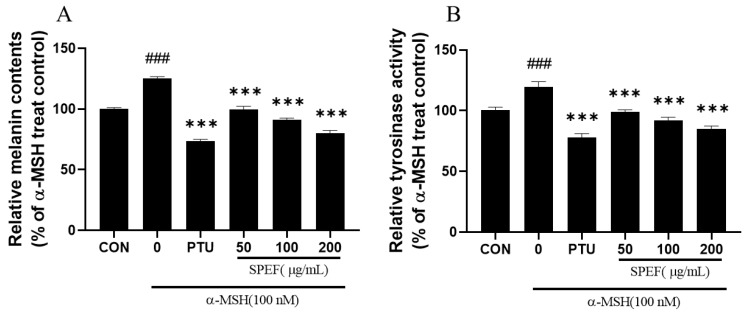
Effects of SPEF on the melanin contents (**A**) and cellular tyrosinase activity (**B**) in α-MSH-induced B16F10 melanoma cells. Values are expressed as mean ± SD in triplicate experiments. ### *p* < 0.001 indicates a significant difference compared to the control group. *** *p* < 0.001 indicates a significant difference compared to the group treated with α-MSH only.

We investigated the effects of SPEF treatment (50–200 μg/mL) on melanin synthesis and intracellular tyrosinase activity in α-MSH (100 nM)-exposed B16F10 melanoma cells. SPEF treatment effectively inhibited α-MSH-induced melanin overproduction (Figure 2A) and intracellular tyrosinase activity (Figure 2B) in B16F10 melanoma cells. Interestingly, the highest concentration (200 μg/mL) of SPEF treatment displayed similar anti-melanogenic activities compared to PTU. These results show that SPEF can effectively reduce acceleration of melanogenesis by inhibiting tyrosinase activity in α-MSH-induced B16F10 melanoma cells.

### 2.4. Effects of SPEF on Cellular Melanogenesis-Related Protein Expressions

**Figure 3:**
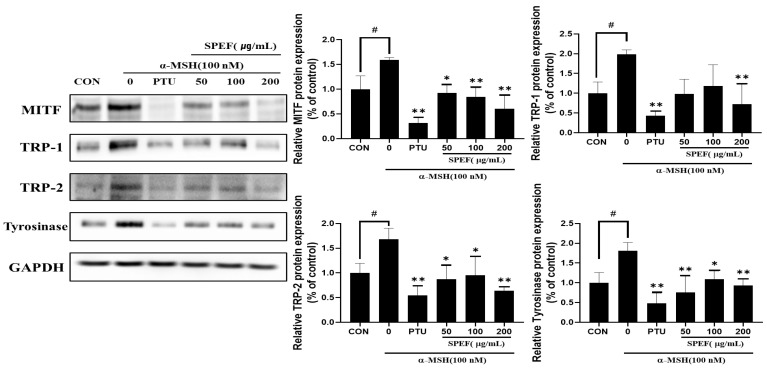
Effects of SPEF on the expression levels of melanogenesis-related proteins in α-MSH-induced B16F10 cells. Values are expressed as mean ± SD in triplicate experiments. # *p* < 0.05 indicate a significant difference compared to the control group. ** *p* < 0.01 and * *p* < 0.05 indicates a significant difference compared to the group treated with α-MSH only.

Melanin formation is closely related to the increment in the expression levels of key melanogenic proteins, including MITF, TRP-1, TRP-2, and tyrosinase. Hence, our study explored the effects of SPEF on MITF, TRP-1, TRP-2, and tyrosinase expressions in α-MSH-treated melanoma cells. Our study showed that α-MSH treatment remarkably increased the expression levels of all the melanogenic proteins; however, SPEF treatment significantly decreased all the protein expression levels in B16F10 cells (Figure 3).

### 2.5. Effects of SPEF on CREB/PKA Signaling Pathway

**Figure 4:**
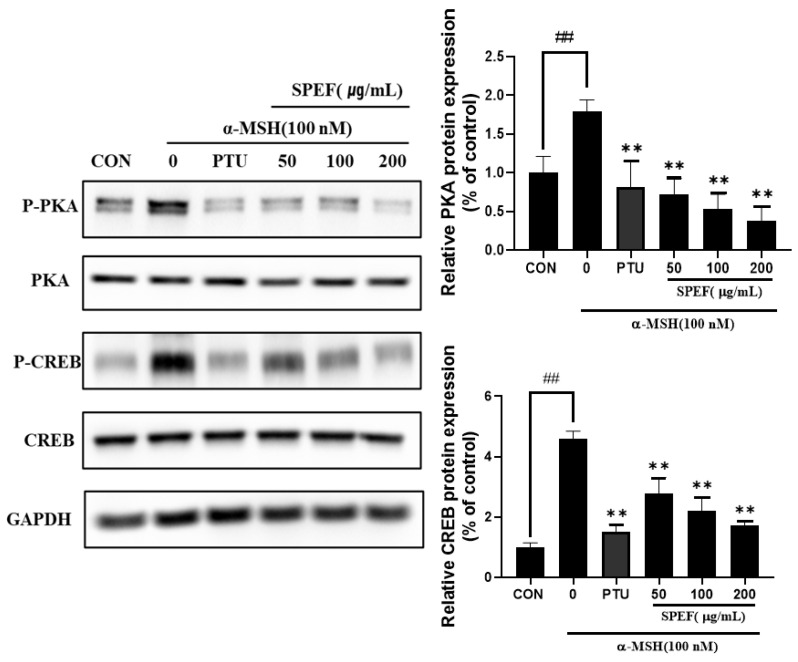
Effects of SPEF on PKA/CREB signaling pathway in α-MSH-induced B16F10 cells. Values are expressed as mean ± SD in triplicate experiments. ## *p* < 0.01 indicate significant differences compared to control group. ** *p* < 0.01 indicates significant difference compared to group treated with α-MSH only.

Our results indicate that SPEF significantly downregulated MITF, tyrosinase, TRP-1, and TRP-2 expression in α-MSH-stimulated B16F10 cells (Figure 3). Recent studies have reported that the activation of PKA and CREB can promote melanin synthesis through the regulation of MITF expression [5,6]. Therefore, we investigated the effects of SPEF on the PKA/CREB signaling pathway to elucidate the underlying mechanisms of SPEF on MITF inhibition. Our results revealed that SPEF effectively normalized α-MSH-stimulated elevation of both the phosphorylation levels of PKA and CREB in murine melanoma cells (Figure 4). These findings demonstrate that the inhibitory effect of SPEF on melanogenesis may be attributed to the downregulation of the PKA/CREB signaling pathway.

### 2.6. Effects of SPEF and PTU on the Toxicity in Zebrafish Embryos

**Figure 5:**
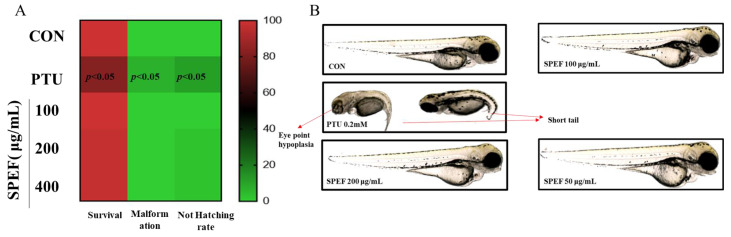
Effect of SPEF and PTU on toxicity in zebrafish embryos. (**A**) Heatmap analysis showing the effects of SPEF and PTU on survival rate, malformation rate, and hatching rate of zebrafish embryos. For survival rate, red indicates higher values (better survival), and green indicates lower values (poorer survival). For malformation rate and hatching rate, green represents better outcomes (lower malformation or higher hatching rates), while red indicates worse outcomes (higher malformation or lower hatching rates). Significant differences (*p* < 0.05) are marked in the heatmap compared to the control group. (**B**) Representative images of morphological changes in zebrafish embryos exposed to SPEF and PTU at 3 days post-fertilization (dpf).

Heatmap analysis illustrates the effects of SPEF and PTU on survival rate, malformation rate, and hatching rate in zebrafish embryos. For survival rate, red indicates higher values (better survival), and green indicates lower values (poorer survival). Conversely, for malformation rate and hatching rate, green represents better outcomes (lower malformation or higher hatching rates), while red indicates worse outcomes (higher malformation or lower hatching rates). SPEF treatment showed no significant effects on these parameters when compared to the control (non-treated) group, indicating its safety. In contrast, PTU treatment exhibited significant toxicity, with marked differences at the *p* < 0.05 level compared to the control group (Figure 5A). Representative images of zebrafish embryos at 3 days post-fertilization (dpf) highlight the morphological changes induced by PTU and SPEF treatments. PTU-treated embryos displayed severe abnormalities, including yolk sac malformation, tail bending, and eye hypoplasia, with 7 ± 6.67% phenotypic changes. On the other hand, embryos treated with SPEF at all tested concentrations showed normal development, comparable to the control group (Figure 5B).

### 2.7. Effects of SPEF on Melanin Synthesis in Zebrafish Embryos

**Figure 6:**
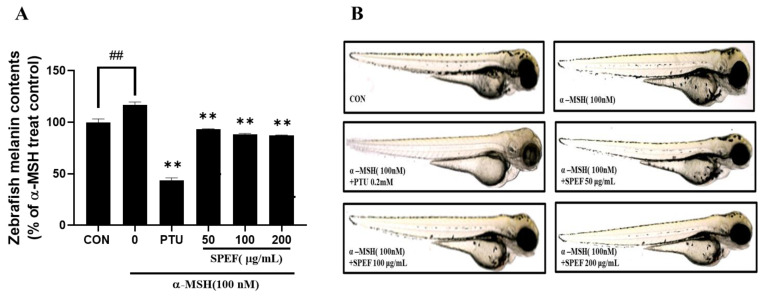
Effects of SPEF on melanin contents in α-MSH-induced zebrafish embryos. Zebrafish larvae were treated with SPEF for 72 h, and PTU was used as a positive control. (**A**) Relative melanin content was calculated through normalizing with control group. (**B**) Photos of zebrafish morphology. SPEF and PTU treatment significantly reduced melanin content in zebrafish embryos. Representative images of the whole body of zebrafish larva captured using a microscope. Values are expressed as mean ± standard deviation (SD), n = 3. ## *p* < 0.01 indicates a significant difference compared to the control group. ** *p* < 0.01 indicates a significant difference compared to the group treated with α-MSH only.

The in vivo anti-melanogenic effects of SPEF were investigated in α-MSH-induced zebrafish model. Zebrafish embryos were exposed to 50, 100, and 200 μg/mL of SPEF and PTU (0.2 mM) for 72 h. SPEF treatment significantly reduced melanin production in α-MSH-induced zebrafish embryos (Figure 6). This observation was clearly confirmed by the representative images of the zebrafish embryos, suggesting that SPEF also exhibited potent in vivo anti-melanogenic effects consistent with the in vitro results.

### 2.8. LC-Q-TOF-MS/MS Analysis of Ethyl Acetate Fraction Derived from Sargassum Pallidum Extract (SPEF)

**Figure 7:**
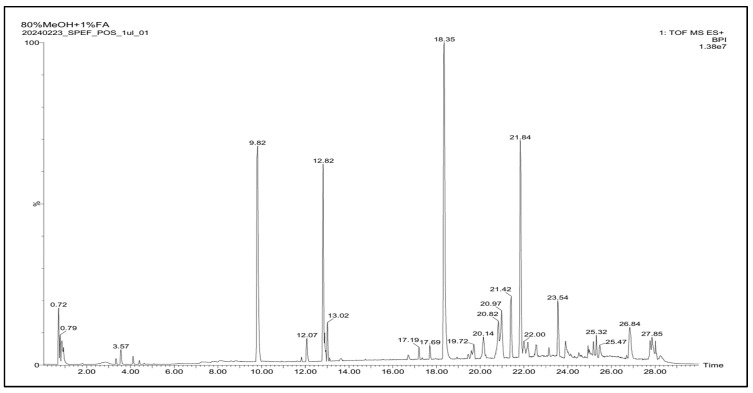
LC-Q-TOF-MS/MS analysis of SPEF.

It is well known that brown algae contain various phenolic compounds, such as terpenoids [12]. To profile the active compounds contained in SPEF, the LC-Q-TOF-MS/MS method was applied. It was found that SPEF contains multiple organic compounds and terpenoids, including aspartate, heptatriene, cardenolide, and sclareol. The chromatogram of SPEF is shown in Figure 7. The detailed profiles of the compounds in SPEF, such as formula, average mass (Da), and transition (*m*/*z*), are given in Table 2. These results suggest that SPEF contains many derivatives from organic compounds, which might be responsible for the skin-brightening activities of SPEF.

## 3. Discussion

Skin, the largest organ of the human body, plays a protective and defensive role against environmental factors such as pathogens, pollutants, and ultraviolet (UV) radiation [17,18]. Especially, melanocytes, responsible for melanin synthesis, protect deeper skin tissues from external damage and subsequent DNA mutations by absorbing and neutralizing the deteriorative effects of UV radiation [1,19]. However, excessive melanin production leads to skin darkening and hyperpigmentation, which can trigger various skin disorders and tumorigenesis. Therefore, the need for the development of anti-melanogenic and skin-brightening agents is still increasing in the pharmaceutical and cosmeceutical industries [20,21]. In this context, many classical skin-brightening agents such as arbutin and PTU have been used; however, various adverse effects and skin toxicities hindered their usage as cosmetic products. Moreover, recent studies have reported the neurodevelopmental toxicity of PTU in an in vivo zebrafish model [22]. Therefore, the development of both effective and non-toxic melanin inhibitors is urgently required.

Natural products, which are compounds or substances derived from living organisms, constitute a diverse array of secondary metabolites. These metabolites are categorized based on their chemical structures and compositions, with notable examples including phenolic compounds, terpenoids, and alkaloids [23,24]. Phenolic compounds, which are characterized by their phenolic hydroxyl groups, exhibit a strong affinity for binding to proteins and macromolecules. They have exhibited various physiological activities, such as antioxidant and anticancer properties, in various cellular and animal studies [25]. Marine algae, which thrive in extreme environmental conditions, are known to produce a wide range of bioactive compounds to resist against outer stress. In particular, many studies have reported that brown algae are rich in active components such as phenolics, fatty acids, and polysaccharides, which exhibit antioxidant, anti-inflammatory, and anti-melanogenic properties [12,13]. *S. pallidum*, a species of marine brown seaweed, has been recognized for its diverse biological activities, which are attributed to its rich array of bioactive compounds [13,14]. In our previous study, we identified that the ethyl acetate fraction derived from *S. pallidum* extract (SPEF) is a promising source of natural antioxidant and anti-inflammatory agents against particulate matter (PM) in human keratinocytes [15]. Based on these findings, we aimed to investigate whether *S. pallidum* could also play a role in suppressing melanogenesis, demonstrating its potential as a source of multifunctional skincare agents. Therefore, we evaluated the skin-brightening effects of SPEF and analyzed the bioactive components of SPEF to identify key compounds responsible for its melanin regulation activities.

Marine algae are known to produce a wide variety of structurally diverse natural products [26]. Many previous studies have reported that *S. pallidum* contains significant amounts of bioactive compounds, contributing to its diverse pharmacological properties [13,14]. In this study, LC-Q-TOF-MS/MS analysis was performed to identify the active components of SPEF which could be associated with potential skin-brightening effects. As the constituents of SPEF, 12 phytochemical compounds were detected and identified. Among these compounds, sclareol, a kind of diterpene, stands out due to its unique bicyclic structure and molecular formula (C_20_H_32_), derived from four isoprene units. Notably, its structure includes two hydroxyl groups (-OH), which contribute to its moderate polarity and underlying biological activities [27,28]. The hydroxyl groups in sclareol can play a critical role in its tyrosinase-inhibitory activity by binding to the active site of tyrosinase, a key enzyme in melanin biosynthesis, thereby blocking melanin overproduction [29]. Hydroxylated compounds like sclareol often exhibit a higher affinity for tyrosinase, which is associated with their effects on the melanogenesis pathway, resulting in the inhibition of melanin synthesis and pigmentation [30]. Therefore, our study speculated that SPEF may inhibit tyrosinase activity and melanin production, which was confirmed by subsequent experiments, including enzyme-substrate response assays, cell-based assays, and a zebrafish model, to portray deeper insights into its skin-brightening potential.

Tyrosinase is a rate-limiting enzyme that plays a pivotal role in melanogenesis, and its overproduction can lead to hyperpigmentation disorders. Therefore, tyrosinase inhibition is a primary strategy for developing skin-brightening agents [31,32]. Firstly, we evaluated the tyrosinase-inhibitory activities of SPE and its fractions (SPHF, SPCF, SPEF, SPBF, and SPWF). Among them, SPEF exhibited the highest tyrosinase activity, with an IC_50_ concentration of 0.03 mg/mL. Melanin is synthesized through the physiological process of tyrosinase-mediated melanogenesis. To investigate whether SPEF could inhibit melanin synthesis, we used α-MSH-stimulated B16F10 melanoma cells, which are widely used in melanogenesis research and effectively reflect the physiological process of melanin production.

Our results demonstrated that SPEF significantly suppressed melanin production and intracellular tyrosinase activity without exhibiting cytotoxicity in α-MSH-induced B16F10 cells. These results were in line with the strong inhibitory effect of SPEF on mushroom tyrosinase, suggesting its potential as a skin-brightening agent.

MITF serves as a central transcription factor regulating melanogenesis [33]. It is well established that α-MSH upregulates MITF expression, which subsequently enhances the expression levels of tyrosinase, TRP-1, and TRP-2, thereby promoting melanin synthesis. Many studies emphasize the importance of MITF regulation as a pivotal factor in the melanogenesis pathway [34]. To further investigate the molecular mechanisms of the inhibitory effects of SPEF on melanogenesis, the expression levels of MITF, TRP, and tyrosinase were analyzed. SPEF significantly suppressed the elevated levels of MITF, tyrosinase, TRP1, and TRP2 expressions in α-MSH-stimulated melanoma cells. These findings suggest that SPEF can reduce tyrosinase activity by downregulating MITF-mediated melanogenic regulators, leading to the inhibition of melanin overproduction.

The PKA signaling pathway is also responsible for melanogenesis, which is promoted by the secretion of α-MSH [35]. The activation of PKA facilitates the phosphorylation of CREB, a crucial transcription factor involved in MITF expression [36]. To elucidate the underlying mechanisms of SPEF inhibiting MITF expression, we examined the phosphorylation levels of PKA and CREB. Our findings demonstrated that SPEF also remarkably suppressed α-MSH-induced upregulation of PKA and CREB, the key regulators of MITF expression. These results altogether indicate that SPEF can inhibit MITF-mediated melanogenic process by modulating PKA/CREB signaling pathway in α-MSH-stimulated B16F10 melanoma cells.

Zebrafish (Danio rerio) has emerged as a valuable in vivo model for evaluating physiological activities, owing to its small size, high fecundity, transparent embryos, cost-effective maintenance, and significant physiological similarities to mammals compared to classic mammalian models [37]. Recently, zebrafish has been increasingly recognized as a robust tool to identify novel brightening agents in phenotype-based research [22,38]. PTU, a widely used brightening agent, is known for its strong melanogenesis-inhibitory properties; however, its developmental toxicities on zebrafish model, such as yolk sac abnormalities, highlight the importance of the development of safe brightening agents [39]. In this regard, the zebrafish model has garnered significant attention as a powerful platform for assessing the toxicity of various functional ingredients and bioactive compounds [15]. Accordingly, the developmental toxicity and anti-melanogenic potential of SPEF were simultaneously evaluated using this model. Various methodologies have been established to quantify melanin levels in zebrafish embryos, including highly sensitive approaches such as Electron Paramagnetic Resonance (EPR) and biochemical assays. EPR is a powerful magnetic resonance technique that detects paramagnetic species with unpaired electrons, making it particularly useful for analyzing melanin and its free radical properties in biological systems. Its application in melanogenesis studies provides precise insights into pigment formation and oxidative states [40]. Meanwhile, biochemical assays of melanin quantify the pigment in a sample through chemical methods, allowing for precise analysis in various biological materials, including hair, tissue samples, and cultured cells [41]. In this study, we adopted a utilizing biochemical method to measure melanin production in zebrafish embryos. As a result, SPEF markedly attenuated α-MSH-induced melanin overproduction and elicited notable depigmentation in zebrafish embryos without inducing morphological abnormalities, thereby demonstrating its superior safety profile compared to PTU. These results are consistent with in vitro cellular experiments, indicating the dual advantages of SPEF as a safe and effective anti-melanogenic agent.

In conclusion, our study firstly showed that SPEF suppressed α-MSH-induced melanogenesis by modulating the PKA/CREB/MITF signaling pathway in B16F10 melanoma cells and a zebrafish model. These results suggest that SPEF can be a promising candidate for the development of skin-brightening agents that can be applied to cosmeceuticals and pharmaceuticals. Further studies are needed to standardize SPEF and explore the interactions between the active compounds and underlying mechanisms of the anti-melanogenic effects.

Further comprehensive studies are required to standardize SPEF and elucidate the interactions between its active constituents and their mechanisms of action. Expanding this research to incorporate human melanocytes and clinical trials will be crucial to reinforce the evidence and validate the potential of SPEF for skin-brightening applications

## 4. Materials and Methods

### 4.1. Preparation of Ethyl Acetate Fraction from SARGASSUM Pallidum Extract (SPEF)

The method for SPEF preparation is described in a previous study [15]. In brief, *Sargassum pallidum* was obtained from Jeju Island, Republic of Korea, and then dried and powdered. The powdered *S. pallidum* was extracted with 70% ethanol (Daejung Chemicals & Metals Co., Ltd., Cheongju, Republic of Korea) for 24 h at room temperature. After filtering the extract using 4 the filtrate obtained was evaporated using a rotary evaporator (Eyela Rotary evaporator N-1000, Tokyo Rikakikai Co., Ltd., Tokyo, Japan) and then freeze-dried to obtain the extract. Subsequently, the 70% ethanol extract (SPE) was dissolved in distilled water and fractionated using solvent-solvent partitioning, resulting in the hexane fraction (SPHF), chloroform fraction (SPCF), ethyl acetate fraction (SPEF), butanol fraction (SPBF), and water fraction (SPWF). The fraction was concentrated and freeze-dried. The fraction from *Sargassum pallidum* extract was stored in the dark at 4 °C for later use.

### 4.2. Liquid Chromatography–Mass Spectrometry (LC-MS/MS) Analysis

To analyze the active ingredients of SPEF, 10 mL of 80% methanol (Daejung Chemicals, Cheongju, Republic of Korea) was added to 1 g of SPEF and centrifuged (12,000 rpm, 10 min), and the supernatant was used for analysis. The supernatant was analyzed using LC-Q-TOF MS (Waters Corp., Milford, MA, USA). WATERS BEH C18 Columm (2.1 mm × 100 mm, 1.7 μm; Waters, Milford, MA, USA) was used to analyze the active ingredients of apple peel extract. The mobile phase was water containing 0.1% formic acid (A) and acetonitrile (B) containing 0.1% formic acid; the flow rate was 0.3 mL/min, and the column temperature was 40 °C. Materials that passed through the column were analyzed using Q-TOF MS–positive electrospray ionization (ESI) mode. Q-TOF MS data were analyzed under the following conditions: mass range: 100~1300 *m*/*z*; capillary: 3 kv; desorbation gas: 600 L/h; desorbation temp: 250°; source temp: 100 °C. Additionally, data processing was performed using Progenesis QI (v.2.4, Waters, Milford, MA, USA) software, and substances were identified using Waters’ linked ChemSpider database.

### 4.3. In Vitro Tyrosinase Inhibitory Assay

The evaluation of the inhibition of tyrosinase activity was performed as previously described [42]. A total of 0.1 M of sodium phosphate buffer (pH 6.8) was mixed with 10 μL samples and after mixing, a tyrosinase enzyme solution in phosphate buffer was added (1500 units/mL). Then, 20 μL of 1.5 mM L-tyrosine was mixed in 96-well plates and incubated at 37 °C for 12 min. The reaction was stopped by incubating on ice for 1 min and the absorbance of the mixture was measured at 490 nm using a microplate reader (SynergyTM HTX Multi-Mode Reader, BioTek, Winooski, VT, USA). The tyrosinase inhibitory activity (%) was counted as follows: inhibition (%) = (1 − B/A) × 100%. A is the absorbance of the blank and B is the absorbance of the sample.

### 4.4. Cell Culture

B16F10 mouse melanoma cells were obtained from American Type Culture Collection (ATCC; Manassas, VA, USA), maintained in Dulbecco’s modified Eagle medium (DMEM, Welgene, Republic of Korea) containing 10% fetal bovine serum (FBS, Welgene, Republic of Korea), 100 U/mL of penicillin, and 100 µg/mL of streptomycin in an incubator with 5% CO_2_ at 37 °C.

### 4.5. Cell Viability

Cell viability assessment was conducted as previously described [43]. Briefly, B16F10 cells were seeded (1 × 10^4^ cells/well) in 24 well culture plates. After seeding, a 24-hour incubation period was followed by sample treatment for 72 h. MTT (3-(4,5-dimethylthiazol-2-yl)-2,5-diphenyltetrazolium bromide) was added to the wells and incubated for 3 h. After removing the supernatants, formazan crystals were dissolved in DMSO, and the absorbance was measured at 540 nm using a microplate reader (SynergyTM HTX Multi-Mode Reader, BioTek, Winooski, VT, USA).

### 4.6. Measurement of Melanin Content

The measurement of melanin content was conducted by modifying a previously described method [44]. In brief, B16F10 cells were seeded (2.5 × 10^4^ cells/well) in 6 well plate and incubated in the presence or absence of 100 nM α-MSH for 1 h. Subsequently, various concentrations of SPEF and PTU were added, and the B16F10 cells were incubated for 72 h. After treatment, the B16F10 cells were washed twice with PBS. Then, 1× trypsin (200 μL) and PBS (800 μL) were added. The cells were collected and centrifuged at 13,000× *g* for 10 min. Next, 1 N NaOH containing 10% DMSO was added. After incubation at 80 °C for 1 h and thorough mixing to solubilize the melanin, the amount of melanin was determined by measuring the absorbance at 475 nm using a microplate reader (SynergyTM HTX Multi-Mode Reader, BioTek, Winooski, VT, USA).

### 4.7. Measurement of Tyrosinase Activity

The measurement of tyrosinase activity was performed using a method derived by modifying a previously described method [44]. In brief, B16F10 cells were seeded (2.5 × 10^4^ cells/well) in a 6-well plate. The cells were treated with 5 nM α-MSH and SPEF. After treatment, the B16F10 cells were washed twice with PBS and lysed in 50 mM sodium phosphate buffer (pH 6.8). Cell lysates were collected by centrifugation at 12,000 rpm for 30 min at 4 °C.

After quantification and standardization, 80 μL of the cell lysate was added to 96-well microtiter plates, and 10 mM L-DOPA was included for incubation at 37 °C for 1 h. After incubation, tyrosinase activity was measured using a spectrophotometer at 492 nm with a microplate reader (SynergyTM HTX Multi-Mode Reader, BioTek, Winooski, VT, USA).

### 4.8. Western Blot Analysis

The B16F10 cells were treated with SPEF and PTU, followed by two cold PBS washes and subsequent harvesting. Afterward, cell homogenization transpired in lysis buffer composed of 20 mM Tris, 5 mM EDTA, 10 mM Na4P2O7, 100 mM NaF, 2 mM Na_3_VO_4_, 1% NP-40, 10 mg/mL Aprotinin, 10 mg/mL Leupeptin, and 1 mM PMSF. Determination of total protein concentrations was executed utilizing a BCATM protein assay kit (Pierce, Rockford, IL, USA). Equally loaded protein samples underwent separation through SDS-PAGE gel electrophoresis and transfer onto nitrocellulose membranes. These membranes were then blocked with 5% nonfat dry milk in TBST (25 mM Tris–HCl, 137 mM NaCl, 2.65 mM KCl, 0.05% Tween 20, pH 7.4) for 1.5 h at room temperature, followed by incubation with primary antibodies: MITF, tyrosinase, TRP-1, TRP-2 p-CREB, CREB, P-PKA, PKA and GAPDH. Subsequent washing with TBST preceded incubation with secondary antibodies for 2 h at room temperature. Visualization of protein bands occurred using a chemiluminescence detection system (FUSION SOLO Vilber Lourmat system, Paris, France).

### 4.9. Maintenance of Parental Zebrafish and Treatment

Zebrafish were procured from a commercial supplier (Sin-yong aquarium, Cheonan, Republic of Korea) and maintained in a 3 L acrylic tank at a temperature of 28.5 °C under a 14:10-hour light–dark cycle. The zebrafish were fed three times a day. Embryos were obtained at a mating ratio of 2 males to 2 females and were induced in the morning by turning on the light. The collection of embryos was completed within 30 min in Petri dishes. Zebrafish embryos were exposed to α-MSH (100 nM) and SPEF (50,100,200 μg/mL), as well as PTU( 0.2 mM) for 3 days.

### 4.10. Phenotype-Based Evaluation in Zebrafish

Animal studies were conducted according to the University Guidelines for Animal Experimentation (Approval number: SCH23-0085). Phenotype-based assessment included an evaluation of the survival rate, hatching rate, and morphological changes. The survival rate, hatching rate, and the impact on the pigmentation of zebrafish were determined at 3 days post-fertilization (dpf). After exposure to SPEF (50,100,200 μg/mL) and PTU (0.2 mM) at 3 dpf, zebrafish embryos were anesthetized with a tricaine methanesulfonate solution (Sigma, St. Louis, MO, USA) and then photographed using the EVOS XL Core Imaging System.

### 4.11. Melanin Contents of Zebrafish Embryos

The melanin contents of zebrafish embryos were determined according to previously described method [45]. In brief, zebrafish embryos at 3 dpf were sonicated in PRO-PREP Protein Extraction Solution (Intron, Seoul, Republic of Korea). After centrifugation, the obtained pellet was dissolved in 500 μL of 1 N NaOH at 100 °C for 30 min. The mixture was vortexed vigorously to solubilize the melanin in the sample. Then, the optical density of the supernatant was measured at 490 nm using a microplate reader (SynergyTM HTX Multi-Mode Reader, BioTek, Winooski, VT, USA).

### 4.12. Statistical Analysis

All the experiments were conducted in triplicate. The data are expressed as the mean ± standard error (SE), and one-way analysis of variance. Analysis of the results was performed using the SPSS statistical program (Version 28, IBM, Armonk, NY, USA). Significant differences (* *p* < 0.05, ** *p* < 0.01, # *p* < 0.05, ## *p* < 0.01) were determined by one-way analysis of variance (ANOVA), complemented by Duncan’s multiple range test.

## Figures and Tables

**Table 1:** Tyrosinase inhibition activity of fraction from *Sargassum pallidum extract*.

Sample	SPE	SPHF	SPCF	SPEF	SPBF	SPWF
Tyrosinase inhibition IC_50_ value (mg/mL)	0.21	1<	0.22	0.03	1<	1<

**Table 2:** Compounds in the LC-Q-TOF-MS/MS profile of SPEF.

NO.	Name	RT (Min)	Formula	Average Mass (Da)	Transition (*m/z*)
1	Bromobenzene	0.72	C_6_H_5_Br	157.008	156.9654
2	Calcium sulfate hemihydrate	0.79	H_2_Ca_2_O	290.297	290.8470
3	Diethyl N-acetyl-3-(ethylsulfanyl) aspartate	12.82	C_12_H_21_NO_5_S	291.364	292.1227
4	Methyl 5-{(3aS,5R,6R,6aS)-5-hydroxy-6-[(1E,3R)-3-hydroxy-1-octen-1-yl]-1,3a,4,5,6,6a-hexahydro-2-pentalenyl} pentanoate	17.19	C_22_H_36_O_4_	364.519	365.2677
5	TEMPO	19.72	C_9_H_19_NO	157.253	158.1539
6	16,17-isopropylidenedioxy-6-methylprogesterone	20.14	C_25_H_36_O_4_	400.551	401.2667
7	Cardanolide	20.82	C_23_H_36_O_2_	344.531	345.2774
8	(5Z)-4-[(1E)-1-Propen-1-yl]-2,3,5-heptatriene	21.84	C_10_H_14_	134.218	135.1164
9	Sclareol	23.54	C_20_H_36_O_2_	308.499	309.2778
10	Chaulmoogric acid	25.32	C_18_H_32_O_2_	280.445	281.2487
11	Cyclohexylundecanoic acid	25.47	C_17_H_32_O_2_	268.435	269.2470
12	Ethephon	27.85	C_2_H_6_ClO_3_P	144.494	144.9819

## Data Availability

All data analyzed during this study are included in this article.
